# Prenatal Fumonisin Exposure Impairs Bone Development via Disturbances in the OC/Leptin and RANKL/RANK/OPG Systems in Weaned Rat Offspring

**DOI:** 10.3390/ijms24108743

**Published:** 2023-05-14

**Authors:** Ewa Tomaszewska, Halyna Rudyk, Siemowit Muszyński, Monika Hułas-Stasiak, Norbert Leszczyński, Maria Mielnik-Błaszczak, Janine Donaldson, Piotr Dobrowolski

**Affiliations:** 1 Department of Animal Physiology, Faculty of Veterinary Medicine, University of Life Sciences in Lublin, 20-950 Lublin, Poland; galusik.77@gmail.com; 2 Laboratory of Feed Additives and Premixtures Control, State Research Control Institute of Veterinary Drugs and Feed Additives, 79000 Lviv, Ukraine; 3 Department of Biophysics, Faculty of Environmental Biology, University of Life Sciences in Lublin, 20-950 Lublin, Poland; siemowit.muszynski@up.lublin.pl; 4 Department of Functional Anatomy and Cytobiology, Faculty of Biology and Biotechnology, Maria Curie-Sklodowska University, 20-033 Lublin, Poland; monhul@o2.pl (M.H.-S.); piotr.dobrowolski@umcs.lublin.pl (P.D.); 5 Department of Agricultural, Forest and Transport Machinery, Faculty of Production Engineering, University of Life Sciences in Lublin, 20-612 Lublin, Poland; norbert.leszczynski@up.lublin.pl; 6 Chair and Department of Developmental Dentistry, Medical University of Lublin, 20-081 Lublin, Poland; mielnikmb@gmail.com; 7 School of Physiology, Faculty of Health Sciences, University of the Witwatersrand, Parktown, Johannesburg 2193, South Africa; janine.donaldson@wits.ac.za

**Keywords:** fumonisin, weaned rats, bone, OPG, RANKL, MMP-8, TIMP-2, VEGF, expression

## Abstract

The goal of the current study was to examine the effects of prenatal exposure to fumonisins (FBs) on bone properties and metabolism in weaned rat offspring divided into groups intoxicated with FBs at either 0 (the 0 FB group), 60 (the 60 FB group), or 90 mg/kg b.w. 0 (the 90 FB group). Female and male offspring exposed to FBs at a dose of 60 mg/kg b.w. had heavier femora. Mechanical bone parameters changed in a sex and FBs dose-dependent manner. Growth hormone and osteoprotegerin decreased in both sexes, regardless of FBs dose. In males osteocalcin decreased, while receptor activator for nuclear factor kappa-Β ligand increased regardless of FBs dose; while in females changes were dose dependent. Leptin decreased in both male FBs-intoxicated groups, bone alkaline phosphatase decreased only in the 60 FB group. Matrix metalloproteinase-8 protein expression increased in both female FBs-intoxicated groups and decreased in male 90 FB group. Osteoprotegerin and tissue inhibitor of metalloproteinases 2 protein expression decreased in males, regardless of FBs dose, while nuclear factor kappa-Β ligand expression increased only in the 90 FB group. The disturbances in bone metabolic processes seemed to result from imbalances in the RANKL/RANK/OPG and the OC/leptin systems.

## 1. Introduction

The importance of good nutrition during pregnancy is well known. However, the daily diet can include supplements [1,2] or other components, which could be toxic and exert harmful effects on fetus development and lead to health problems later in postnatal life, according to the hypothesis of prenatal programming and prenatal origin of diseases [3]. Any poorly stored food could be a source of toxins produced by mold/fungi, especially in certain favorable climates. Fungi, like Fusarium, produce heat-resistant mycotoxins called fumonisins (FBs; types A, B, C and P) [4]. The most frequently occurring FBs of similar toxicity are type B1 and 2 (FB1 and FB2), found in over 50% of food and feed raw material, in a ratio of about 3:1 of FB1:FB2 [5,6,7]. Both FB1 and FB2 inhibit sphingosine N-acyltransferase [8], while FB2 also inhibits the protein serine/threonine phosphatase [9]. FBs disturb sphingolipid metabolism and cause nephrotoxicity, hepatocarcinogenesis, immunosuppression and neurotoxicity [10,11,12,13]. Moreover, Aspergillus niger, a mold commonly found in soil, water, vegetation and fecal matter also produces FBs, leading to higher consumption of mycotoxin-contaminated feed than expected [14]. Non-species specific hepatic or kidney symptoms of FBs intoxication are observed in non-rodent and rodent species and are dependent on animal sex and age, as well as on fumonisin dose and route of administration [12,15,16,17]. Species-specific, organ-specific symptoms of FBs intoxication have also been observed in horses and swine [18].

FBs are also potentially hazardous to humans [8,19,20,21], according to the International Agency for Research on Cancer (IARC) [22]. The total amount of FBs acceptable in human foods and animal feed is controlled by the European Commission and the U.S. Food and Drug Administration (FDA) in the USA [23,24,25]. Moldy food, contaminated with FBs (maize, dried figs, grapes, raisins, wine, coffee and some plants), has been found in China, South America, Africa and Europe [14,22,26,27,28,29,30,31]. Worldwide, human FBs exposure, through moldy maize or animals products, accounts even 354.0 μg/kg body weight per day [32,33,34]. FBs cross the placental barrier and exert many negative effects on fetus development depending on the FBs dose, duration of exposure and time of the exposure [35,36,37]. Infants, young children and even pregnant women are at risk of consuming food contaminated with FBs [38,39,40,41]. Humans are unknowingly exposed to FBs through food intake due to mycotoxins are difficult to monitor continuously, and a tools to assess the risk are poor [42,43,44]. Prenatal FBs exposure results not only in altered prenatal development, disturbances in the structure and function of bone and the gastrointestinal tract (liver, intestine, including enteric nervous system), impaired hematopoiesis and inflammation in skeletal muscles, it also leads to endocrine alterations resulting in disproportional development and increased risk of diabetes mellitus [12,37,45,46,47,48,49,50,51]. Considering FBs-exposure results in various health problems, including imbalances in the collagenous and non-collagenous bone proteins involved in bone turnover, it seems reasonable to further investigate the effects of prenatal FBs exposure on bone metabolism, which is important in the development of the whole organism and its functioning. Changes in the expression of bone non-collagenous proteins has been described in newborns [46], but there is no data concerning all these proteins in the next stages of life, where postnatal mammal development differs from that which occurs prenatally, which is more determined by the mother’s metabolism.

Therefore, the objective of this study was to determine the effects of maternal FBs exposure on bone morphological traits, mechanical properties, trabecular bone histomorphometry, and bone metabolism regulated by osteocalcin/leptin (OC/leptin) and RANKL/RANK/OPG systems. The expression of osteoprotegerin (OPG) and receptor activator of nuclear factor kappa-Β ligand (RANKL), matrix metalloproteinase-8 (MMP-8), tissue inhibitor of metalloproteinases 2 (TIMP-2), vascular endothelial growth factor (VEGF), and cartilage oligomeric matrix protein (COMP) in the bone tissue of both male and female weaned rats was determined. In doing so, the present study examined (i) the expression of the above-mentioned proteins in bone tissue via western blot, (ii) the changes in mechanical properties and trabecular morphology after maternal FBs exposure, (iii) the determination of OPG and RANKL and other hormones in blood serum. All these measurements should provide fundamental information regarding the outcomes of prenatal FBs exposure on postnatal bone metabolism upon weaning.

## 2. Results

### 2.1. Body Weight and Morphometrical Bone Parameters

**Figure 1:**
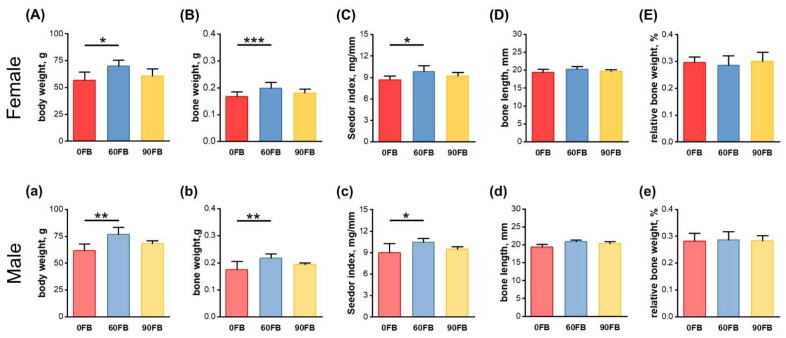
The effect of exposure of pregnant Wistar rat dams to fumonisins on (**A**,**a**) offspring weight at weaning, and the geometric parameters of female (**B**–**E**) and male (**b**–**e**) offspring femora: (**B**,**b**) bone weight; (**C**,**c**) the Seedor index; (**D**,**d**) bone weight; (**E**,**e**) relative bone weight. 0 FB—the control group; 60 FB—the group of offspring of pregnant rats receiving during pregnancy fumonisins at the dose of 60 FB/kg b.w.; 90 FB—the group of offspring of pregnant rats receiving during pregnancy fumonisins at the dose of 90 FB/kg b.w. Data are mean values ± SD. Statistical significance: * *p* < 0.05; ** *p* < 0.01; *** *p* < 0.001.

At weaning, females and males exposed to FBs at a dose of 60 mg/kg b.w. weighed significantly more (Figure 1A,a) and had heavier femora (Figure 1B,b), with a higher Seedor index (Figure 1C,c), compared to the 0 FB group. No other changes were observed.

### 2.2. Mechanical Bone Parameters

**Figure 2:**
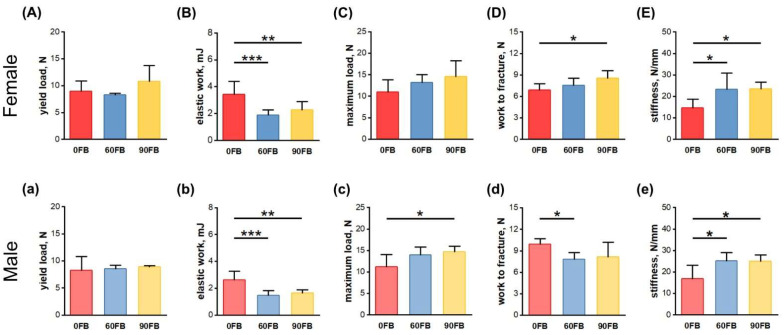
The effect of exposure of pregnant Wistar rat dams to fumonisins on the mechanical parameters of female (**A**–**E**) and male (**a**–**e**) offspring femora at weaning: (**A**,**a**) yield load; (**B**,**b**) elastic work; (**C**,**c**) maximum load; (**D**,**d**) work to fracture; (**E**,**e**) stiffness. 0 FB—the control group; 60 FB—the group of offspring of pregnant rats receiving during pregnancy fumonisins at the dose of 60 FB/kg b.w.; 90 FB—the group of offspring of pregnant rats receiving during pregnancy fumonisins at the dose of 90 FB/kg b.w. Data are mean values ± SD. Statistical significance: * *p* < 0.05; ** *p* < 0.01; *** *p* < 0.001.

In the femora of the female rats, elastic work values decreased in both prenatally FBs-exposed groups (Figure 2B), and work to fracture values were significantly increased in the 90 FB group (Figure 2D) compared to the values of these parameters in the 0 FB group. Moreover, femora stiffness was increased following FBs exposure, regardless of dose (Figure 2E).

In males, femora maximum load was significantly increased in the 90 FB group (Figure 2c), while work to fracture values were lower in the 60 FB group, compared to these parameters in the 0 FB group (Figure 2d). Moreover, elastic work of the male femora were significantly increased (Figure 2b) and stiffness significantly increased (Figure 2e) compared to the 0 FB group, irrespective of FBs dose. No other changes were observed.

### 2.3. Hormonal Analysis

**Figure 3:**
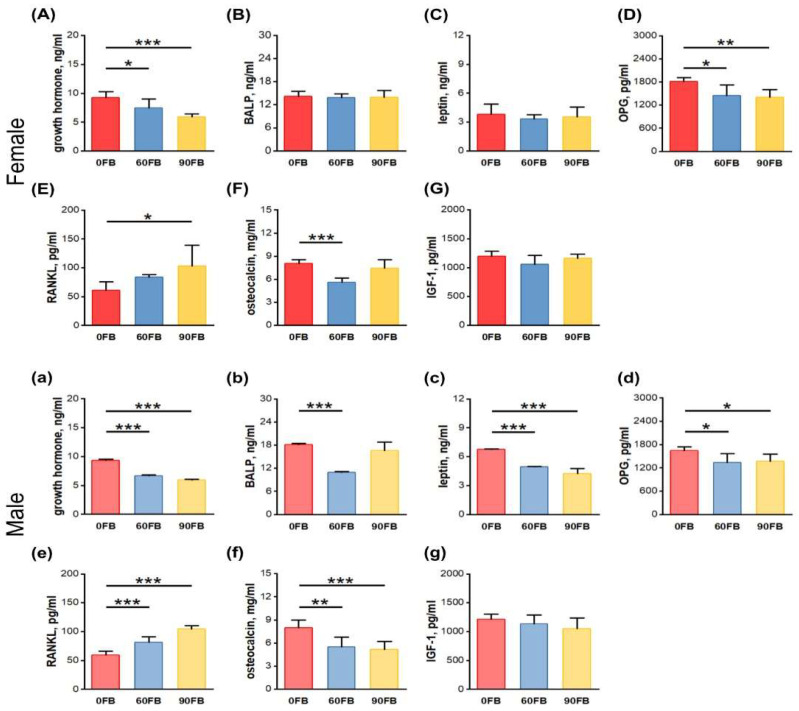
The effect of exposure of pregnant Wistar rat dams to fumonisins on female (**A**–**G**) and male (**a**–**g**) offspring serum levels of: (**A**,**a**) growth hormone; (**B**,**b**) bone alkaline phosphatase (BALP); (**C**,**c**) leptin; (**D**,**d**) osteoprotegerin (OPG); (**E**,**e**) nuclear factor-kappa-Β ligand (RANKL); (**F**,**f**) osteocalcin; (**G**,**g**) insulin-like growth factor-1 (IGF-1). 0 FB—the control group; 60 FB—the group of offspring of pregnant rats receiving during pregnancy fumonisins at the dose of 60 FB/kg b.w.; 90 FB—the group of offspring of pregnant rats receiving during pregnancy fumonisins at the dose of 90 FB/kg b.w. Data are mean values ± SD. Statistical significance: * *p* < 0.05; ** *p* < 0.01; *** *p* < 0.001.

Serum concentrations of growth hormone (GH) (Figure 3A,a) and osteoprotegerin (OPG) (Figure 3 D,d) decreased in both male and female rats following prenatal FBs exposure, regardless of FBs dose. In males, the serum concentration of osteoprotegerin (OC) decreased (Figure 3d), while that of receptor activator of nuclear factor-kappa-Β ligand (RANKL) increased (Figure 3e) following FBs exposure, compared to control rats, regardless of FBs dose. Serum concentrations of RANKL were increased in the 90 FB group of female rats (Figure 3E), while osteocalcin (OC) concentrations were decreased in the 60 FB group of female rats (Figure 3F), compared to that of the 0 FB group. Moreover, serum leptin concentrations decreased significantly in all males exposed to FBs (Figure 3c) and bone alkaline phosphatase (BALP) concentrations decreased only in the 60 FB group (Figure 3b), compared to the control. No other changes were observed.

### 2.4. Protein Expression

**Figure 4:**
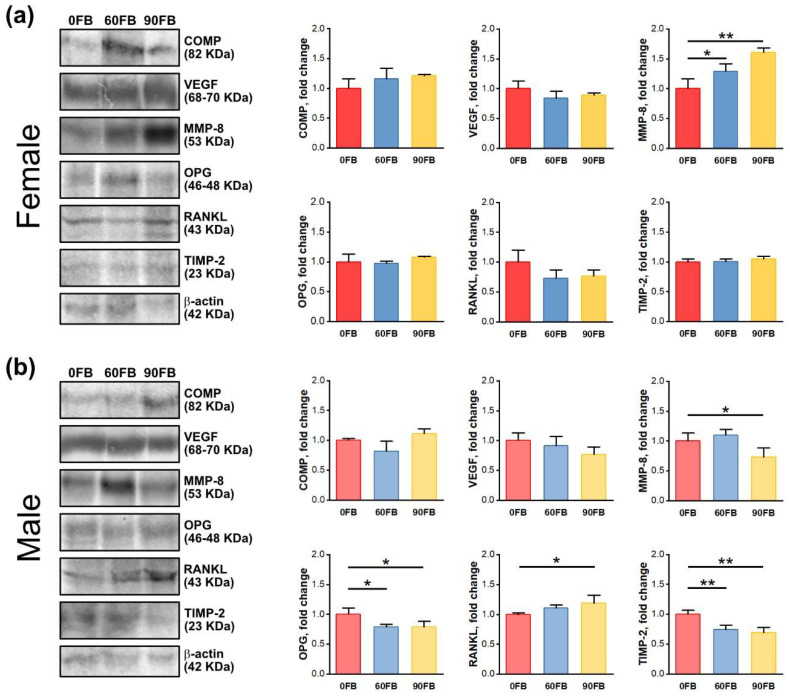
The effect of exposure of pregnant Wistar rat dams to fumonisins on COMP, VEGF, MMP-8, OPG, RANKL and TIMP-2 protein expression in female (**a**) and male (**b**) offspring femora. The protein levels are normalized to the corresponding β-actin levels and expressed as the fold change relative to the amount present in 0 FB control group. 0 FB—the control group; 60 FB—the group of offspring of pregnant rats receiving during pregnancy fumonisins at the dose of 60 FB/kg b.w.; 90 FB—the group of offspring of pregnant rats receiving during pregnancy fumonisins at the dose of 90 FB/kg b.w. Data are mean values ± SD (*n* = 3). Uncropped original Western blot membranes can be found in Supplementary Figure S1. Statistical significance: * *p* < 0.05; ** *p* < 0.01.

Metalloproteinase-8 (MMP-8) expression increased in all females exposed to FBs (Figure 4a), while it was significantly decreased in males in the 90 FB group, compared to the 0 FB group (Figure 4b). Osteoprotegerin (OPG) and tissue inhibitor of metalloproteinase-2 (TIMP-2) protein expression was decreased in males exposed to FB, regardless of FBs dose, while RANKL expression increased only in the 90 FB group (Figure 4b), compared to the control rats.

### 2.5. Trabecular Bone Morphology

**Figure 5:**
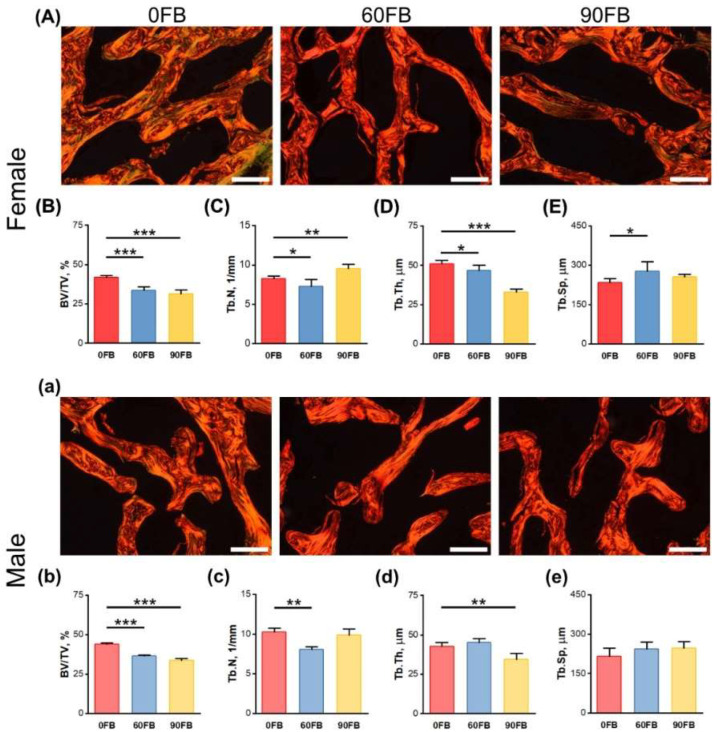
The effect of exposure of pregnant Wistar rat dams to fumonisins on the basal morphological parameters of trabecular bone of the distal diaphysis of femora of female (**A**–**E**) and male (**a**–**e**) offspring. (**A**,**a**) Representative images of the formaldehyde-fixed, PSR-stained sections of the femoral distal diaphysis showing trabecular bone. Observations were carried out using polarized light microscopy. All scale bars show 40 µm; (**B**,**b**) bone volume fraction (BV/TV); (**C**,**c**) trabecular number (Tb.N); (**D**,**d**) trabecular thickness (Tb.Th); (**E**,**e**) trabecular spacing (Tb.Sp). 0 FB—the control group; 60 FB—the group of offspring of pregnant rats receiving during pregnancy fumonisins at the dose of 60 FB/kg b.w.; 90 FB—the group of offspring of pregnant rats receiving during pregnancy fumonisins at the dose of 90 FB/kg b.w. Data are mean values ± SD. Statistical significance: * *p* < 0.05; ** *p* < 0.01; *** *p* < 0.001.

Formaldehyde-fixed, PSR-stained sections of the femoral distal epiphysis were analyzed using images acquired through polarized light microscopy (Figure 5A,a). A decrease in bone volume fraction (BV/TV) was observed in rats of both sexes after maternal FBs administration (Figure 5B,b). The decrease in trabecular number (Tb.N) in the 60 FB group was observed regardless of the sex of the offspring, while in the 90 FB group the decrease of Tb.N was only observed in females (Figure 5C,c). The decrease of trabecular thickness (Tb.Th) was observed in the 90 FB group regardless of sex, while in the 60 FB group only in females (Figure 5D,d). The trabecular spacing (Tb.Sp) only decreased in the 90 FB group of females (Figure 5E,e).

## 3. Discussion

The FBs-induced inhibition of ceramide synthases results in disturbances in the metabolism of sphingolipids and affects several signaling systems important for cellular growth and differentiation, resulting in reduced feed efficiency and weight gain [15]. Prenatal FBs exposure triggers neural tube defects and other developmental alterations due to the interaction between mycotoxin, genetic, epigenetic, and metabolic factors, in a dose-dependent manner [52]. In the last few years there have been a few animal studies focused on bone metabolism after postnatal or prenatal FBs exposure, which have reported endosteal resorption and weakening of the bone, linked with thinning of the bone wall [12,45,46,53,54]. Moreover, previous studies have shown a probable risk for the occurrence of diabetes mellitus in weaned offspring of mothers exposed to FBs during pregnancy, due to hormonal dysregulation [48].

In terms of bone development and the maintenance of proper bone mass, there are two main processes involved, bone modeling and remodeling. In early life (infant, childhood and adolescence), bone modeling predominates resulting in the lengthening and widening of the bones which is responsible for achievement of the proper size, shape and weight of the skeleton. After reaching peak bone mass, bone remodeling occurs on a continuous basis, involving two opposite processes of bone formation and resorption, that remain in equilibrium to maintain bone mass. Bone formation and resorption are also important for the repair of bone damage and disintegration, and play a main role in calcium-phosphate homeostasis within the body [55,56].

Bone metabolic processes involve two systems: the OC/insulin/leptin system and the OPG/RANK/RANKL system [57,58,59,60]. OC is an indicator of bone mineralization and osteoblastic bone formation and is produced by osteoblasts and osteocytes and also acts as a stimulator of testosterone production. OC is speculated to be analogous to leptin, increasing the sensitivity of adipose tissue to insulin and limiting fat mass, as well as influencing insulin synthesis in the pancreas. Mice lacking OC accumulate body fat and eventually develop glucose intolerance [57,61,62]. Elevated glucose concentrations block the osteocalcin gene promoter in humans [63]. There exists interrelationship between OC, obesity and insulin resistance [58,64]. Leptin, via the hypothalamus, has been shown to increases OC release from osteoblasts, leading to the modulation of the glucose-insulin relationship and weight homeostasis. Leptin is a pleiotropic hormone produced by adipocytes, that participates in modulation of the immune response, reproduction, lipolysis and angiogenesis [59]. Leptin’s centrally-mediated, stimulatory action enhances osteocalcin secretion by osteoblasts, without insulin participation [65]. It presents presenting well-documented beneficial leptin’s effect on bone architecture and energy homeostasis [59].

The current study involved weaned rats which have been shown to be underweight at birth, regardless of the sex, and overweight upon weaning, as previously reported [48]. The weaned rats in the current study displayed increased insulin concentrations after exposure to FBs, mainly at a dose of 90 mg/kg b.w., without any changes in the glucose concentration; while after exposure to FBs at a dose of 60 mg/kg b.w., weaned rats showed enhanced glucose concentrations, without alterations in insulin concentration [48].

The current study also showed decreased leptin and OC concentrations, regardless of FBs dose in males, without any changes in leptin concentration in females, which were dose-dependent. However, increased glucose concentrations were only observed after exposure to FBs at a dose of 60 mg/kg b.w. [48]. This could be a result of insulin insensitivity induced by the decrease in OC, as was proven by Reinehr et al. [58]. On the other hand, future studies should investigate whether prenatal exposure to higher FBs doses results in the impairment of insulin receptors and lower concentrations of OC and leptin in weaned males. It is well-known that leptin reduces RANKL [66]. Thus, weaned male rats showed the decreased concentration of leptin and enhanced RANKL concentration. Additionally, a decrease in OPG was observed.

RANKL and OPG are cytokines involved in the bone turnover process [67]. OPG is released by osteoblasts, dendritic cells, lymphocytes, spleen, bone marrow, heart, liver and even kidneys [68], while RANKL is expressed by osteoblasts, osteoclasts and primary mesenchymal cells surrounding cartilage and chondrocytes, as well as on endothelial cells, lymph nodes, thymus, and lung and is expressed at low levels in a variety of other tissues, including the spleen and bone marrow [69]. RANKL enhances the differentiation of osteoclasts from precursors and intensifies their activity and viability through the inhibition of apoptosis, thereby promoting bone resorption. The RANKL/RANK/OPG system plays a central role in bone loss and osteoporosis. Osteoclasts are stimulated by the interaction of RANKL with the RANK receptor on their surface. This process is inhibited when OPG binds RANKL. Imbalances between RANKL and OPG results in the destruction of bone [67]. A previous study, on the effects of prenatal exposure to FBs (at the same doses as presented here) on bone development in newborns, showed changes in OPG and RANKL expression in a dose- and sex-dependent manner [45].

The current study showed not only an increase in RANKL concentration, but also a decrease in OPG concentration, which could result in bone loss. Western blot analysis showed that these changes were sex-dependent. The expression of both cytokines in the bones of weaned, male rats (distal epiphysis and metaphysis) was in line with the changes observed in the blood serum. On the other hand, one should remember that these cytokines are also released by many other cells, as mentioned above. In the case of weaned, male rats, prenatally exposed to FB, a decreased expression of MMP-8 was also observed. The role of MMP-8 and FBs in degradation of the extracellular matrix (ECM) has been previously discussed [46]. Western blot analysis showed that MMP-8 expression decreased significantly following the 90 mg FBs dose in both sexes of newborns [46]. MMP-8 is an enzyme, which degrades type I, II, III and IV collagen in bone and cartilage during development and bone remodeling and increases cytokines and initiates inflammation and osteoarthritis [70,71]. Cartilage oligomeric matrix protein (COMP), found primarily in cartilage, is linked with binding calcium and responsible for the interaction of collagen with other matrix proteins. It stimulates collagen fibril formation during overall body growth [72]. A previous study showed enhanced COMP expression in males following prenatal FBs exposure at a dose of 60 mg/kg b.w., indicating enhanced matrix turnover and an apparent attempt at repair (as mentioned above) [46].

The current study on weaned rats showed no changes in COMP expression, regardless of the sex or FBs dose. However, with time there were sex-dependent alterations in MMP-8 expression. Whether this means that degradation of the ECM was more intensive in females than in males, should be further investigated. Western blot analysis in the current study also showed a decrease in TIMP-2 expression in weaned, male rats prenatally exposed to FBs, regardless of FBs dose. The role of TIMP-2 as a natural inhibitor of MMPs in the development of bone in newborn offspring exposed to FBs, has been previously discussed [46], with TIMP-2 expression being dose- and sex-dependent. The current study showed that with time TIMP-2 expression becomes only sex-dependent. This is in line with previous data reporting dose and compartment-dependent changes in TIMP-2 expression, that indicate sex-dependent differences in bone matrix turnover processes in newborn rats [46]. TIMP-2 is also a natural inhibitor of angiogenesis, while vascular endothelial growth factor (VEGF) is pro-angiogenic. VEGF also stimulates MMPs expression to facilitate bone vascularization and indirectly, bone growth [73]. VEFG acts through Flk1/VEGFR2-Src-PI3K-Akt-eNOS-NO pathway resulting in regulation of cell’s survival, permeability and migration [46,74].

Prenatal exposure to FBs did not influence VEGF expression in weaned rats, which was contrary to previous effects observed in newborns, where VEGF expression in bone tissue increased following FBs exposure, in a sex- and dose dependent manner. The current study showed that weaned rats of both sexes had heavier bones after prenatal FBs exposure at 60 mg/kg b.w., which was in line with the changes in final body weight and the Seedor index (which is related to bone density) [75], even though serum GH was reduced. On the other hand, it should be highlighted that these rats had elevated serum glucose, as mentioned above. GH secretion varies under various conditions. Not only does age, sex, body mass index and the distribution of adipose tissue influence GH action, but it has also been proven that overweight, hyperinsulinemia or hyperglycemia inhibits GH secretion [76,77].

GH acts via insulin like-growth factor-1 (IGF-1), which further affects bone alkaline phosphatase (BALP) and the process of bone mineralization, involving signaling pathways such as Wnt, BMP2, FGF and IGFBP/IGF [78]. The current study showed a decrease in BALP in a dose- and sex-dependent manner, which was associated with a reduction in GH, but without any changes in IGF-1. Bone mineralization is additionally restricted by vitamin D, which is also involved in the modulation of BALP expression, via the above mentioned mechanism [78]. It should be mentioned that myco-intoxication results in vitamin D deficiency [79].

The correct bone metabolic processes are important for the maintenance of proper bone structure and mechanical strength, and can be modified by genetic, environmental, nutritional or hormonal factors, as well as bone geometry [80,81]. The current study was performed on one strain of rats that were all fed the same basal diet and housed under the same environmental conditions. Thus, maternal exposure to various FBs doses (0, 60 or 90 mg/kg b.w.) was the main factor affecting bone characteristics (mechanical properties and basal trabecular morphology) in the current study. The maternal FBs exposure affected postnatal bone development in a dose- and sex-dependent manner, and these observations were in line with other previous reports [12,16,82,83,84].

A previous study showed reduced bone strength in newborns prenatally exposed to FBs, caused by an increase in immature collagen, which was associated with intense bone turnover, leading to disturbed bone development and function, in a sex- and dose-dependent manner [45]. The current study confirmed these previous sex- and dose-dependent effects of prenatal exposure to FBs, however, an increase in maximal load, fracture load and stiffness of the femora was also observed, in a dose- and sex-dependent manner. It also confirmed previous results involving the expression of various bone proteins in particular bone compartments, highlighting the different effects of FBs on trabecular bone (femoral trabeculae of distal part) and on the mid-shaft of the long bone. This mechanism should be further investigated.

Despite numerous strengths of our study on weaned rats, such as comprehensive analysis of bone mechanical strength, quantitative western blot analyses of expression of proteins involved in bone turnover, histomorphometry of trabecular bone, and hormonal blood and biomarkers of bone turnover analysis, the current study has some limitations. These include no immunolocalization of bone proteins studied and the absence of analysis of their gene expression. However, these limitations do not diminish the novelty of our work. To the best of our knowledge, this is the first study to show the involvement of both the OC/leptin and RANKL/RANK/OPG systems in the maintenance of bone metabolic homeostasis in weaned rats prenatally exposed to FBs, which seems to be sex-dependent. Nevertheless, it seems that both the direct and indirect effects of FBs intoxication on bone development are complicated and should be further examined at different life stages and metabolic pathways.

## 4. Materials and Methods

The study was approved by the Institutional Ethics Committee of State Scientific Research Control Institute of Veterinary Medicinal Products and Feed Additives in Lviv, Ukraine (#132 676-Adm/08/2020). The experiment was carried out in compliance with the ARRIVE guidelines.

### 4.1. Fumonisins Preparation and Quantification

FBs were produced in vitro on a maize grain with the use of *F. moniliforme*, as previously described [12,47]. The contaminated grains showed the 3:1 (73% to 27%) ratio of FB1 and FB2. FBs were EtOH extracted from ground maize grains, quantified using an RIDASCREEN Fumonisin ELISA kit (R3401, R-Biopharm, Darmstadt, Germany) and concentrated by evaporation to a FBs stock solution (100 mg FB1 + FB2/ml) [12]. During the experiment, the FBs extract stock was diluted in 0.9% saline solution to yield the necessary concentrations in 0.5 mL.

### 4.2. Animals and Experimental Design

Pregnant Wistar rats at the age of five weeks (*n* = 18) were individually housed in polypropylene cages (the dimensions of 380 × 200 × 590 mm), at a temperature of 21 ± 3 °C and humidity of 55 ± 5%, with a 12 h/12 h day/night cycle. A standard diet for laboratory rats was offered ad libitum, with unlimited access to water. After an acclimatization period, all pregnant dams were randomly allocated to one of three groups (*n* = 6 in each group): a control group (the 0 FB) or one of two FBs-intoxicated groups: receiving FBs at a dose of 60 mg FB/kg b.w. (the 60 FB) or 90 mg FB/kg b.w. (the 90 FB), respectively. The 90 mg FB/kg b.w. dose, equal to 1/10 of the established LD50 dose, is sufficient to induce sub-clinical intoxication in adolescent rats, while the 60 mg dose, equal to 1/15 of the established LD50 value, did not trigger subclinical or clinical signs in adolescent rats [16,85]. Both doses resulted in higher BW at weaning, with probable insulin resistance, noted mainly after exposure to the of 60 mg/kg b.w. dose [48]. The two doses are routinely used in developmental studies (omitting the first 6 days of pregnancy) carried out at the State Scientific Research Control Institute of Veterinary Medicinal Products and Feed Additives in Lviv, Ukraine [12,16,45,46,47,85]. On the basis of daily measurements of the weight of individual pregnant dams, the FBs mixture, diluted to the dose of 60 or 90 mg FB/kg b.w. the from FBs extract stock in 0.5 mL of 0.9% saline, was given intragastrically daily from the 7th day of pregnancy to parturition. The control pregnant animals were administered a saline solution at the same volume. All pregnant females were under veterinarian care. After natural parturition, all newborn offspring were allocated to the following groups, according to the treatment received by their mothers: 0 FB, 60 FB or 90 FB groups and were kept together with their lactating mothers. On postnatal day 28, two weaned offspring from each dam (one male and one female, *n* = 6 males and *n* = 6 females in total) were weighed and euthanized (CO_2_ inhalation). Immediately after euthanasia (the loss of animals’ respiratory activity), the blood was collected by cardiac puncture and placed into tube with silica cloth activator (BD Vacutainer Systems, Plymouth, UK). The serum samples were prepared through centrifugation of coagulated blood at (1300× *g* for 10 min at 18 °C). The samples of serum were aliquoted into polypropylene tubes and frozen at −20 °C until the assays were performed. Right after euthanasia femora were collected and cleaned. The left femora were immediately frozen in liquid nitrogen and stored at −80 °C, until they were subjected to western blot analysis, while the right femora, after the measurements of length and weight, were individually packed and stored frozen at −20 °C until they were examined for mechanical strength and subsequent analysis of trabecular bone morphometry.

### 4.3. Mechanical Testing

A three-point bending test was performed using a Zwick/Roell Z005 universal testing machine (Zwick-Roell GmbH & Co., Ulm, Germany) equipped with a 50 N load cell. Bones were loaded with a constant load rate of 2 mm/min until fracture. From the recorded load-deformation curves, the following bone mechanical properties were determined: yield load, elastic work, maximum load, work to fracture, and stiffness [86].

### 4.4. Trabecular Bone Morphology

After mechanical testing distal sections of bone (including epiphysis, metaphysis, and part of the midshaft) were fixed in buffered 4% formaldehyde and then decalcified in decalcifying solution (Decalcifier II, Leica Biosystems, Wetzlar, Germany) and embedded in paraffin. Sections (4-μm thick) were cut using a microtome (HM360, Microm, Walldorf, Germany) and stained with Picrosirius red (PSR) staining to assess the morphology of trabecular bone [87]. Stained sections of the distal metaphysis of the femur were observed and photographed using polarized light microscopy (CX43, Olympus, Tokyo, Japan). Obtained images were analysed to assess the basic histomorphometry of the trabecular bone using the public domain ImageJ software (version 1.51 k) [88]. Determined microarchitectural descriptors included bone volume fraction (BV/TV), trabecular thickness (Tb.Th), trabecular number (Tb.N), and trabecular spacing (Tb.Sp) [89].

### 4.5. Western Blot

The left femora that were stored at −80 °C were removed from the freezer and placed were briefly in liquid nitrogen. Next, they were placed in prechilled mortar and pulverized using a pestle until powdered. The powdered bone tissue was transferred to a microtube for addition of ice-cold RIPA lysis buffer containing protease inhibitor cocktail (S8820, Sigma-Aldrich Merck KGaA, Darmstadt, Germany). The Pierce BCA Protein Assay Kit (Thermo Fisher Scientific, Wilmington, DE, USA) was used to determine protein content. Obtained supernatants were aliquoted and stored at −80 °C. Samples containing 80 µg of protein were separated by 12% SDS-PAGE and then transferred to PVDF membranes (Immobilon-P, Sigma-Aldrich, St. Louis, MO, USA). After the transfer, the membranes were blocked with 3% non-fat dry milk in TBS for 1 h and incubated overnight at 4 °C to an appropriate primary antibody: Rb pAb cartilage oligomeric matrix protein (COMP, E-AB-14886, Elabscience, Wuhan, China, dilution 1:1000), Rb polyclonal vascular endothelial growth factor (VEGF, orb191500, Biorbyt, Cambridge, UK, dilution 1:1000), Rb pAb to osteoprotegerin (OPG; ab73400, Abcam, Cambridge, UK, dilution 1:1000), Rb mAb to matrix metalloproteinase 8 (MMP-8; ab81286, Abcam, Cambridge, UK, dilution 1:1000); Rb mAb to receptor activator for nuclear factor kappa-Β ligand (RANKL; E-AB-30151, Elabscience, Wuhan, China, dilution 1:1000), Rb mAb to tissue inhibitor of metalloproteinases 2 (TIMP-2; DF7008, Affinity Biosciences, Jiangsu, China, dilution 1:1000). Alkaline phosphatase-conjugated goat anti-Rb IgG H&L (ab97048, Abcam, Cambridge, UK, dilution 1:10,000) was used as a secondary anti-body. A Rb pAb anti-β-actin (AF7018, Affinity Biosciences, Zhenjiang, China, dilution 1:1000) was used as the loading control. Immunoreactive proteins were visualized using standard alkaline phosphatase visualization procedure in NBT/BCIP (11681451001, Roche, Basel, Switzerland). The membranes were densitometrically quantified and normalized to their corresponding β-actin bands. The quantitative analysis of intensity of protein bands was performed using “Gel Analysis” option in ImageJ software [88]. Uncropped original Western blot membranes can be found in Supplementary Figure S1.

### 4.6. Hormonal Analysis

Blood serum concentrations of growth hormone (GH), bone alkaline phosphatase (BALP), leptin, osteoprotegerin (OPG), receptor activator of nuclear factor kappa B ligand (RANKL), osteocalcin, and insulin-like growth factor-1 (IGF-1) were determined using commercial rat-specific enzyme-linked immunosorbent assay (ELISA) kits (E-EL-R0029, E-EL-R1109, E-EL-R0582, E-EL-R0050, E-EL-R0841, E-EL-R0243, and E-EL-R0010, respectively, Elabscience, Houston, TX, USA) according to the manufacturer’s protocols. The intra-assay CV for all assays was below 7%. Samples were analyzed in duplicate using a Benchmark Plus microplate spectrophotometer (Bio-Rad Laboratories, Inc., Hercules, CA, USA). Results were calculated using standard curves created in individual tests.

### 4.7. Statistical Analysis

Statistical analysis was conducted using Statistica 13.1 software (TIBCO Software Inc., Palo Alto, CA, USA). A normal distribution of data was tested using the Shapiro–Wilk test. For normally distributed data, a one-way ANOVA with Dunnett’s post-hoc test was used to compare FBs groups with the control (the 0 FB group). Non-parametric data were analyzed using a Kruskal–Wallis ANOVA with Dunn’s multiple comparisons test. For all tests, a *p*-value < 0.05 was considered as statistically significant.

## 5. Conclusions

It can be concluded that the negative effects of prenatal FBs exposure on postnatal bone development are dose-, sex- and bone compartment-dependent. It seems that the FBs-induced disturbances in bone metabolic processes resulted from imbalances in the RANKL/RANK/OPG system, involving the OC/leptin system.

## Data Availability

The data presented in this study are available on request from the corresponding author.

## Supplementary Materials

The following supporting information can be downloaded at: https://www.mdpi.com/article/10.3390/ijms24108743/s1.
