# Supplementary material for: Prenatal Fumonisin Exposure Impairs Bone Development via Disturbances in the OC/Leptin and RANKL/RANK/OPG Systems in Weaned Rat Offspring
Source: Int J Mol Sci. 2023 May 14;24(10):8743. doi: 10.3390/ijms24108743 (PMC10217822; doi:10.3390/ijms24108743)
Supplement: Supplementary file 1 [file ijms-24-08743-s001.zip › ijms-2361928-supplementary.pdf]

Prenatal Fumonisin Exposure Impairs Bone Development via Disturbances in the OC/Leptin and RANKL/RANK/OPG Systems in Weaned Rat Offspring

Ewa Tomaszewska, Halyna Rudyk, Siemowit Muszyński, Monika Hułas-Stasiak, Norbert Leszczyński, Maria Mielnik-Błaszczak, Janine Donaldson, Piotr Dobrowolski

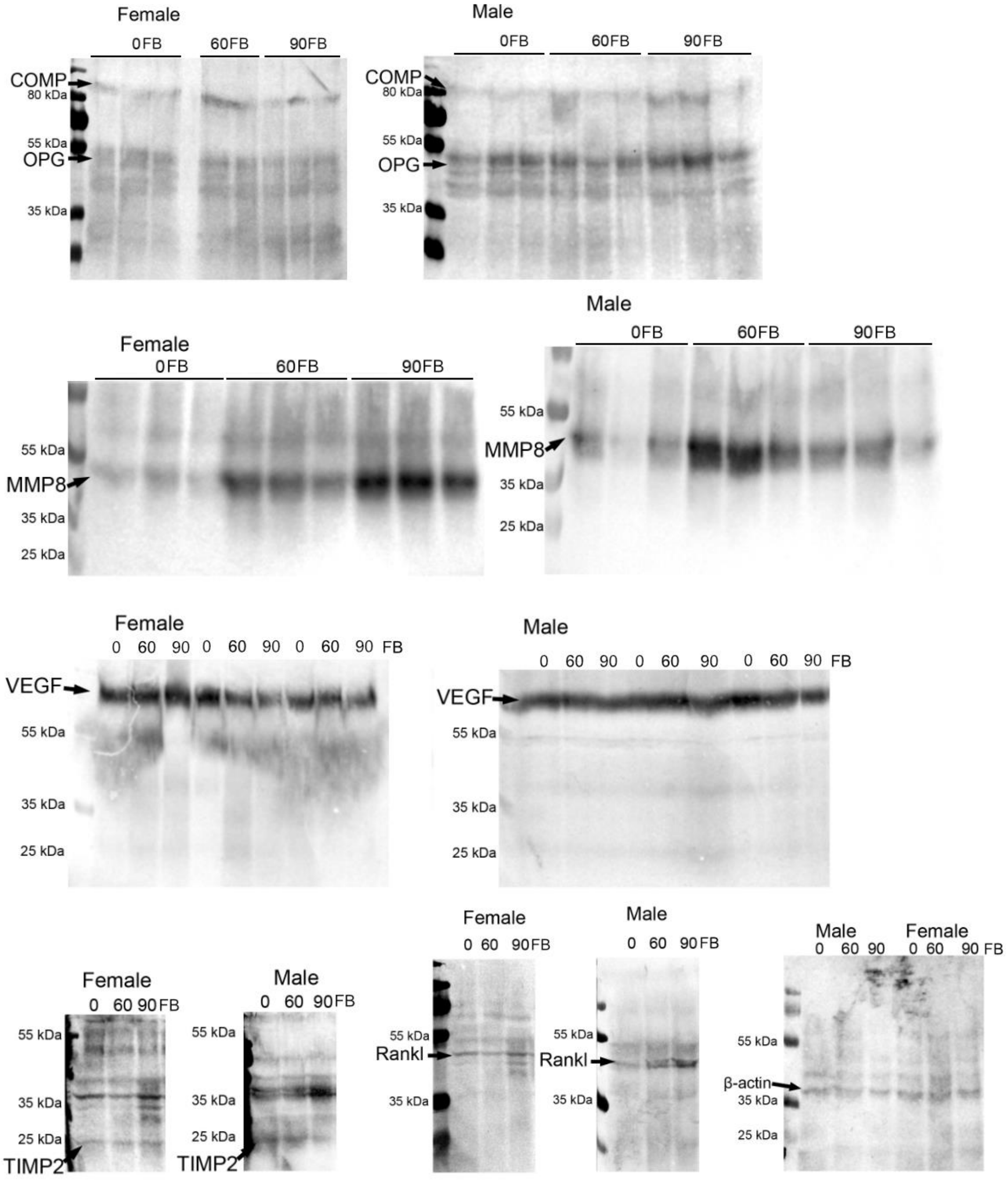

Figure S1: Uncropped original Western blot membranes.
